# Assessment of social skills of adolescents victims of mistreatment

**DOI:** 10.1192/j.eurpsy.2023.1499

**Published:** 2023-07-19

**Authors:** A. Carneiro, D. R. Molini Alvejonas, D. Cardilli Dias

**Affiliations:** 1 Speech Therapy, Physiotherapy and Occupational Therapy - FMUSP, University of São Paulo, São Paulo, Brazil

## Abstract

**Introduction:**

Between January and May 2022, more than 78,248 complaints were registered in Brazil, involving children and adolescents in situations of abuse. (Ministry of Women, Family and Human Rights, 2022). Childhood maltreatment includes all forms of physical, emotional, sexual abuse or neglect. Literature has shown that individuals who are victims of abuse have more difficulties with social skills than their peers.

**Objectives:**

Quantitatively evaluate the social skills of adolescent victims of abuse.

**Methods:**

Six adolescents between 12 and 17 years old participated in the study. The assessment of social communication skills was performed using the Social Skills Rating Scale (SSRS) protocol. At the time of data analysis, the following variables were taken into account: gender of the participants and type of abuse suffered, as stated in their medical records.

**Results:**

Male participants showed a better overall performance in social skills than female participants. However, this second group scored higher on assertiveness and empathy skills. Regarding the type of abuse, in both genders, victims of sexual abuse and neglect showed better results than those who suffered only neglect. The results are described in more detail in images 1 and 2.

**Table 1.** Female group results

	Overall score SSRS	Empathy	Self-control	Responsability	Assertiveness
Expected scores	25 – 32	7 – 9,81	6 -10	7- 10	3 – 5
Average of the results obtained (n = 3)	19,66	5,66	5,66	5,66	2,66
Average of the results obtained from neglect victims (n = 2)	17	4	5	4,5	3,5
Average of the results obtained from sexual abuse and neglect victims (n = 1)	25	9	7	8	1

**Table 2.** Male group results

	Overall score SSRS	Empathy	Self-control	Responsability	Assertiveness
Expected scores	23 – 31	6 – 9	6 - 9	7 – 9	2 - 5
Average of the results obtained (n = 3)	23,66	4,66	7,66	8,66	1,33
Average of the results obtained from neglect victims (n = 1 )	25	3	10	8	2
Average of the results obtained from sexual abuse and neglect victims (n = 2)	23	5,5	8	7,5	2

**Conclusions:**

Adolescents who are victims of abuse have considerable difficulties with social skills, which can impact the performance of their basic day-to-day activities. More studies about the impacts of mistreatments on the development of social skills are needed.

**Disclosure of Interest:**

None Declared

